# Acute Stroke Biomarkers: Are We There Yet?

**DOI:** 10.3389/fneur.2021.619721

**Published:** 2021-02-05

**Authors:** Marie Dagonnier, Geoffrey A. Donnan, Stephen M. Davis, Helen M. Dewey, David W. Howells

**Affiliations:** ^1^Stroke Division, Melbourne Brain Centre, The Florey Institute of Neuroscience and Mental Health, Melbourne, VIC, Australia; ^2^Department of Neurology, Ambroise Paré Hospital, Mons, Belgium; ^3^Melbourne Brain Centre at the Royal Melbourne Hospital and University of Melbourne, Melbourne, VIC, Australia; ^4^Eastern Health Clinical School, Monash University, Melbourne, VIC, Australia; ^5^Faculty of Health, School of Medicine, University of Tasmania, Hobart, TAS, Australia

**Keywords:** stroke, biomarker, review, microarray, acute

## Abstract

**Background:** Distinguishing between stroke subtypes and knowing the time of stroke onset are critical in clinical practice. Thrombolysis and thrombectomy are very effective treatments in selected patients with acute ischemic stroke. Neuroimaging helps decide who should be treated and how they should be treated but is expensive, not always available and can have contraindications. These limitations contribute to the under use of these reperfusion therapies.

**Aim:** An alternative approach in acute stroke diagnosis is to identify blood biomarkers which reflect the body's response to the damage caused by the different types of stroke. Specific blood biomarkers capable of differentiating ischemic from hemorrhagic stroke and mimics, identifying large vessel occlusion and capable of predicting stroke onset time would expedite diagnosis and increase eligibility for reperfusion therapies.

**Summary of Review:** To date, measurements of candidate biomarkers have usually occurred beyond the time window for thrombolysis. Nevertheless, some candidate markers of brain tissue damage, particularly the highly abundant glial structural proteins like GFAP and S100β and the matrix protein MMP-9 offer promising results. Grouping of biomarkers in panels can offer additional specificity and sensitivity for ischemic stroke diagnosis. Unbiased “omics” approaches have great potential for biomarker identification because of greater gene, protein, and metabolite coverage but seem unlikely to be the detection methodology of choice because of their inherent cost.

**Conclusion:** To date, despite the evolution of the techniques used in their evaluation, no individual candidate or multimarker panel has proven to have adequate performance for use in an acute clinical setting where decisions about an individual patient are being made. Timing of biomarker measurement, particularly early when decision making is most important, requires urgent and systematic study.

## Biomarkers

Use of the term “biomarker” describes measures of biological function was first seen in Medline is 1977 and has exploded in the last decade (1). A US National Institutes of Health working group defined a biomarker as: “a characteristic that is objectively measured and evaluated as an indicator of normal biological processes, pathogenic processes, or pharmacologic responses to a therapeutic intervention” (2). While the term “biomarker” can include clinical or imaging measurements, it is usually reserved for describing molecules found in bodily fluids (1).

Biomarkers such as cardiac troponin, creatine kinase, or D-dimer are used in practice in the emergency department for the diagnosis and early management of the life-threatening conditions including myocardial infarction or pulmonary embolism. Indeed, D-dimer measurements are used for the exclusion of a diagnosis of pulmonary embolism with a sensitivity of 96%. A negative D-dimer test will virtually rule out thromboembolism (3). Cardiac troponin (and especially the I isoform) is used routinely to diagnose myocardial infarction with a sensitivity of more than 90% for a cut off value of 0.04 ng/ml (4).

Other biomarkers are used as tools for disease stating (e.g., carcinoembryonic antigen-125 for cancers), for classification of disease severity (e.g., blood prostate-specific antigen concentration to indicate prostate cancer growth and metastasis), to assess disease prognosis (e.g., measurement of tumor shrinkage) or to aid therapeutic monitoring (e.g., blood cholesterol concentrations during therapy to reduce the risk of heart disease) (2).

## The Need For Acute Stroke Biomarkers

Five interventions improve outcome in patients with ischemic stroke. These are thrombolysis with recombinant tissue plasminogen activator (rt-PA) (5), aspirin given within 48 h (6), management of the patients within a dedicated stroke unit (7), hemicraniectomy (8), and more recently endovascular clot retrieval (9).

Thrombolysis is currently recommended for IS patients that present within 4.5 h of stroke onset. Advanced neuroimaging allows extension of this time window up to 9 h and inclusion of patients that wake up with stroke symptoms if salvageable brain tissue can be identified (10, 11). Nevertheless, thrombolysis is disappointingly infrequent in patients with acute ischemic stroke. Indeed, <10% of ischemic stroke patients receive this therapy in most centers and no more than a third in the best performing centers (12–16). The main reasons for this underuse are uncertainty about stroke type, how long the ischemia has been present diagnosis and the associated risks of cerebral bleeding (17–21).

Thrombectomy is currently recommended in IS patients (after or independently from rt-PA) with evidence of large vessel proximal anterior circulation occlusion and within 6 (or 24 h with advanced imaging selection) of symptoms onset (9, 22–24). This revolutionary treatment is unfortunately not in more widespread use than thrombolysis as it is estimated that fewer than 10% of acute IS patients would meet the eligibility criteria and not all stroke centers have sufficient resources and expertise to deliver this therapy (25, 26).

Brain imaging currently plays a critical biomarker role in acute stroke management as it is the only proven way to differentiate ischemic from hemorrhagic stroke. Advanced perfusion imaging can also be used to help select patients that might benefit from rt-PA or thrombectomy under specific circumstances (10, 11, 23, 24). Nevertheless, imaging cost, availability, contraindications, as well as the level of expertise required to interpret advanced imaging results, restricts the global use of reperfusion therapies.

Other less expensive more and accessible stroke biomarkers detected in the blood would be an important addition to the stroke clinician's armory.

An ideal stroke biomarker(s) should be able, with high specificity and sensitivity, to differentiate hemorrhagic and ischemic stroke (and clearly distinguish them from stroke mimics). They should predict stroke prognosis, facilitate therapeutic stratification and therapeutic monitoring, for example by indicating risk of hemorrhagic transformation after stroke or after rt-PA treatment. Moreover, if repeated measures can be made in a clinically useful time frame, specific stroke biomarkers could act as a “stroke clock” to aid in assessing time of stroke onset to increase the number of IS able to benefit from treatment with rt-PA, especially those who wake-up with stroke.

With the advent of mechanical thrombectomy, brain imaging with vascular sequences has become a *de facto* standard in the management of an acute stroke. Nevertheless, a biomarker that provides the same information would facilitate and fasten the access to therapies. It would have the potential to aid early identification and pre-hospital stratification of ischemic stroke patients. Indeed, biomarker stratification of the different classes of stroke patients in a pre-hospital setting would facilitate directing them to a hospital where thrombectomy is performed without losing crucial time by performing brain imaging in the nearest hospital and then transferring the patient to the comprehensive stroke center. It is known that substantial delays of 110–128 min are associated with secondary transfer vs. the direct approach (27).

Over 150 candidate stroke biomarkers have been studied for roles ranging from diagnosis to long term prognosis (28–35).

**Figure 1:**
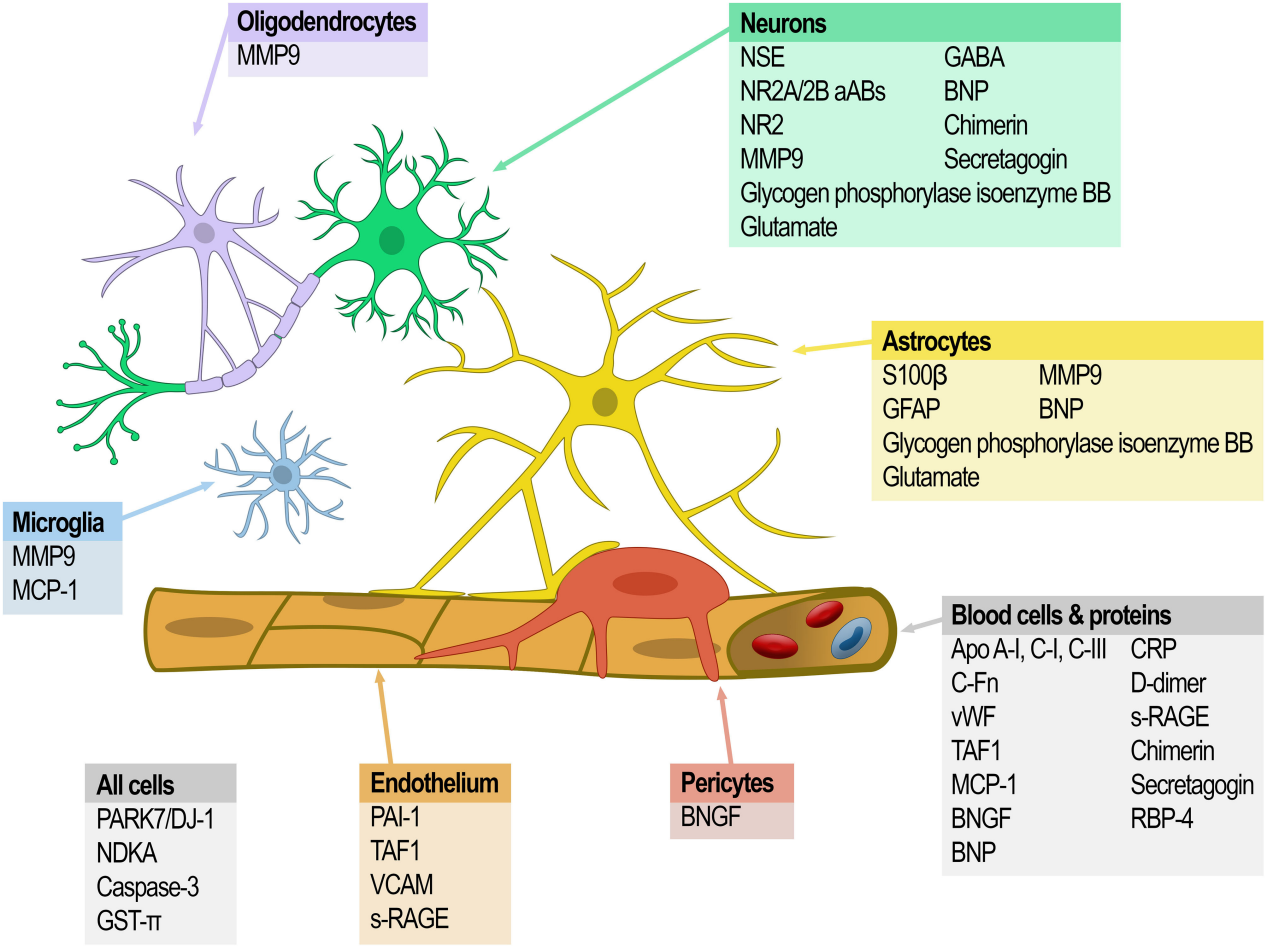
Sources of the major candidate biomarkers.

The following literature review highlights those biomarkers with the potential to have an impact in the acute clinical setting, especially with regard to reperfusion therapy. Moreover, in this acute context, the review has been focused on studies using blood as a substrate for biomarker research because of the ease with which this biological fluid can be accessed in the emergency setting. Table 1 summarizes the most relevant results of this review. Table 2 highlights the main clinical uses ascribed to the potential biomarkers and Figure 1 illustrates the sources of the major candidate biomarkers.

**Table 1:** Summary of the most relevant studies and results of stroke biomarkers.

**Biomarker**	**Function tested**	**Timing of sampling**	**Sensitivity (%)**	**Specificity (%)**	**Cut off value**	***n***	**References**
S100B	Differentiation between IS and ICH	Within 6 h of symptom onset	95.7	70.4	67 pg/ml	71 IS and 46 ICH	(36)
	Differentiation between stroke and mimics	24 h after symptom onset	94.4	31.8	0.0415 ng/ml	31 IS and 22 mimics	(37)
	Risk of hemorrhagic transformation after rt-PA	Within 6 h of symptom onset	82	46	>0.23 g/l	275 rt-PA treated IS	(38)
	Risk of malignant oedema	At 8, 12, 16, 20, and 24 h after symptom onset	75 (at 12 h) 94 (at 24 h)	80 (at 12 h) 83 (at 24 h)	>0.35 g/l (at 12 h) >1.03 g/l (at 24 h)	16 malignant, 35 non-malignant	(39)
GFAP	Differentiation between IS and ICH	Within 4.5 h of symptom onset	84.2	96.3	2.9 ng/l	163 IS, 39 ICH and 3 mimics	(40)
	Differentiation between IS and ICH	Between 2 and 6 h of symptom onset	86	76.9	0.7 ng/ml	65 IS and 43 ICH	(41)
	Differentiation between IS and ICH	Within 4.5 h of symptom onset	61	96	34 ng/ml	79 IS and 45 ICH	(42)
	Differentiation between IS and ICH	Within 6 h of symptom onset	77.8	94.2	0.03 g/l	146 I and 46 ICH	(43)
	Differentiation between IS, ICH and mimics	Within 6 h of symptom onset	91	97	0.43 ng/ml	121 IS, 34 ICH, 31 mimics, 5 SAH, and 79 controls	(44)
NSE	Favorable outcome after rt-PA	Within 4.5 h of symptom onset	77.1	59.4	13.90 ng/ml	67 rt-PA treated IS	(45)
MMP-9	Risk of hemorrhagic transformation after rt-PA	Within 3 h of symptom onset	92	74	>140 ng/ml	134 rt-PA treated IS	(46)
NR2A/2B aAbs	Differentiation between stroke and controls	Within 3 h of symptom onset	95	97	2.0 μg/l	31 IS, 56 TIAs, and 255 controls	(47)
NR2	Differentiation between stroke, mimics and controls	Within 72 h of symptom onset	92	96	1.0 μg/l	101 IS, 91 non-stroke and 52 controls	(48)
Apo C-III	Differentiation between IS and ICH	Within 6 h of symptom onset	94	87	36	16 IS and 15 ICH	(49)
Apo C-I	Differentiation between IS and ICH	Within 6 h of symptom onset	94	73	60	16 IS and 15 ICH	(49)
Apo B	Differentiation between stroke and controls	After a period of overnight fasting for 12 h	96	94	144 mg/dl	50 strokes and 50 controls	(50)
Apo A-I	Differentiation between stroke and controls	After a period of overnight fasting for 12 h	88	86	114 mg/dl	50 strokes and 50 controls	(50)
Apo B/Apo A-I	Differentiation between stroke and controls	After a period of overnight fasting for 12 h	98	96	1.2	50 strokes and 50 controls	(50)
PARK 7	Differentiation between stroke and controls	On admission (median of 17 h after symptom onset)	AUC = 0.897; OR = 1.087		–	72 strokes and 78 controls	(51)
NDKA	Differentiation between stroke and controls	On admission (median of 17 h after symptom onset)	AUC = 0.462; OR = 0.882		–	72 strokes and 78 controls	(51)
Glycogen phosphorylase isoenzyme BB	Differentiation between stroke and controls	Within 12 h of symptom onset	93	93	7.0 ng/ml	172 IS and 133 controls	(52)
c-Fn	Risk of hemorrhagic transformation after rt-PA	Within 3 h of symptom onset	100	60	3.6 μg/ml	27 rt-PA treated IS	(46)
	Risk of malignant oedema	On admission (mean time of 6–7 h after symptom onset)	90	100	>16.6 μg/ml	40 malignant and 35 non-malignant	(53)
PAI-1 and TAFI	Risk of hemorrhagic transformation after rt-PA	Within 3 h of symptom onset	75	97.6	PAI-1 <21.4 ng/mL and TAFI >180%	77 rt-PA treated IS	(54)
Glutamate	Risk of early neurological deterioration	On admission (mean time of 9–10 h after symptom onset)	81	87	>200 μmol/l	27 progressing and 86 non-progressing lacunar strokes	(55)
GABA	Risk of early neurological deterioration	On admission (mean time of 9–10 h after symptom onset)	96	94	<240 nmol/l	27 progressing and 86 non-progressing lacunar strokes	(55)
Combination of S100B, BNGF, vWF, MMP-9, and MCP-1	Differentiation between stroke and controls	Within 12 h of symptom onset	91	97	–	223 strokes and 214 controls	(56)
Combination of S100B, vWF, MMP-9, and VCAM	Differentiation between stroke and controls	Within 24 h of symptom onset	90	90	–	65 IS and 157 controls	(57)
Combination of S100B, MMP-9, D-dimer, BNP, and CRP	Differentiation between stroke and controls	Within 6 h of symptom onset	81	70	–	130 patients with focal neurological deficit	(58)
Combination of S100B, MMP-9, D-dimer, and BNP	Differentiation between stroke and controls	Within 24 h of symptom onset	86	37	–	1,100 patients with focal neurological deficit	(59)
Combination of MMP-9, D-Dimer, sRAGE, caspase-3, chimerin, and secretagogin	Differentiation between stroke and mimics	Within 24 h of symptom onset	Overall accuracy of the model: 0.91		–	915 strokes and 90 mimics	(60)
S100B and sRAGE	Differentiation between IS and ICH	Within 3 and 6 h of symptom onset	22.7	80.2	S100B >96 pg/ml and sRAGE <0.97 ng/ml	776 IS and 139 ICH	(61)
GFAP and RBP4	Differentiation between IS and ICH	Within 6 h of symptom onset	–	100	GFAP <0.07 ng/ml and RBP4 >61 lg/mL	38 IS and 28 ICH	(62)
Panel of 22 genes	Differentiation between stroke and controls	<24, 24–48, >48 h after symptom onset	78	80	–	20 IS and 20 controls	(63)
Panel of 18 genes	Differentiation between stroke and controls	Within 3 h, at 5 h and at 24 h after symptom onset	With accuracy in 66% within 3 h, 86% at 5 h and 100% at 24 h		–	15 IS and 8 controls	(64)
Panel of 23 genes	Differentiation between stroke etiologies	Within 3 h, at 5 h and at 25 h after symptom onset	95.2	95.2	–	15 IS	(65)
Panel of 40 genes	Differentiation between cardio-embolic and large vessel strokes	At 3 h, at 5 h and at 25 h after symptom onset	>90	>90	–	76 IS	(66)
Panel of 34 genes	Differentiation between TIA and patient with CVD	From 9 to 68 h (mean 35 h) after symptom onset	100	100	–	26 TIAs and 26 controls	(67)
Panel of 26 genes	Differentiation between IS or TIA and controls	Within 72 h of symptom onset	89	89	–	94 IS, 26 TIAs, and 44 controls	(68)
Panel of 41 genes	Differentiation between lacunar and non-lacunar strokes	Within 72 h of symptom onset	>90	>90	–	30 lacunar and 86 non-lacunar strokes	(69)
GST-π	Discrimination between early (<3 h) and late (3 h) presentation of stroke	Within 3 h and after 3 h of symptom onset	AUC = 0.79; OR = 10		17.7 μg/l	103 IS and 132 controls	(70)

**Table 2:** Main clinical uses and their linked potential biomarkers.

Differentiation between stroke and controls	S100B NSE NR2A/2B aAbs Apo B, Apo A-I, and Apo B/Apo A-I ratio CRP P-Selectin Homocysteine BNP D-dimer Combination of S100B, MMP-9, vWF, BNGF, and MCP-1 Combination of S100B, MMP-9, vWF, and VCAM Combination of S100B, MMP-9, D-dimer, BNP, and CRP Combination of S100B, MMP-9, D-dimer, and BNP Combination of MMP-9, D-Dimer, sRAGE, caspase-3, chimerin, and secretagogin Panel of genes
Differentiation between IS and ICH	S100B GFAP Apo C-I and Apo C-III BNP S100B and sRAGE GFAP and RBP4
Risk of hemorrhage after rt-PA	S100B NSE MMP-9 c-Fn PAI-1 and TAFI
Correlation with hemorrhage volume	GFAP
Correlation with stroke severity and infarct size	S100B NSE MMP-9
Correlation with favorable neurological outcome	NSE
Acting as a stroke clock	NSE NR2 GST-π PARK7 NDKA
Risk of early neurological deterioration	MMP-9 Glutamate IL-6 TNF-α ICAM-1
Risk of malignant oedema	S100B MMP-9 c-Fn
Stroke etiology	Panel of genes

## Candidate Diagnostic Biomarkers

### S100B

S100B, a glial protein, highly specific to nervous tissue, was one of the first molecules suggested as a candidate to aid IS diagnosis. Hill and colleagues reported a specificity of more than 95% for S100B measured on the first day of admission in 28 non-consecutive stroke patients but the measurement had poor sensitivity (71). More recently, Zhou et al. reported that measuring S100B within the first 6 h of stroke helped differentiate IS from ICH (sensitivity of 95.7%, specificity of 70.4%, using a cut-off of 67 pg/ml) (36). Unfortunately, these results were not substantiated by Gonzalez-García's 2012 study where S100B, measured between 8 and 48 h of symptom onset was significantly elevated in stroke compared to controls but failed to differentiate between IS and ICH and did not correlate with stroke severity on admission (72) despite other studies suggesting that S100B concentrations correlated with stroke severity and size of infarction (73–75).

Serum S100B concentration measured 24 h after symptom onset is significantly higher in stroke patients (posterior circulation IS or infratentorial ICH, no distinction was made in the analysis) than in controls or in patients with vertigo from non-vascular causes (37). However, in addition to prolonged and delayed release into the blood after stroke, S100B levels are also increased in other neurological pathologies such as traumatic brain injuries and extracranial malignancies (76, 77).

Never-the-less, elevated S100B concentration (>0.23 g/l) has been associated with hemorrhage risk due to rt-PA treatment (specificity of 82%, sensitivity 46%) (38) suggesting with further work, this biomarker may have some utility.

### GFAP

GFAP (glial fibrillary acidic protein), another glial protein specific to astrocytes (78), is the best candidate to date for differentiating hemorrhage and ischemic stroke. Based on detection of delayed GFAP release in patients with ischemic stroke (maximum concentration reached 2–4 days after ischemic stroke onset), Foerch and his team studied this molecule in different clinical settings and showed promising results (79–81). In a multicenter clinical study of 205 patients (163 with IS, 39 with ICH and three stroke mimics) diagnostic accuracy was high for differentiating intracerebral hemorrhage from ischemic stroke by GFAP immunoassay on a single blood sample obtained within 4.5 h of symptom onset. Using GFAP cut-off of 2.9 ng/l provided a specificity of 96.3% and a sensitivity of 84.2% for distinguishing ICH, IS and stroke mimics (40). In addition, the levels of GFAP were shown to be correlated with the hemorrhage volume (40, 79, 80).

Several studies have confirmed the potential for serum GFAP to distinguish IS and ICH. Xiong et al. showed that the GFAP concentration in blood collected within 2–6 h after symptom onset was significantly higher in ICH (*n* = 43) than IS (*n* = 65) patients, with 86 and 76.9% sensitivity and specificity of, respectively, using a cut-off point of 0.7 ng/ml) (41). Ren et al. replicated these findings using a GFAP cut off value of 0.34 ng/ml with 61% sensitivity and 96% specificity in a 4.5 h time window from symptoms onset (42). A later study measuring serum GFAP in 46 ICH and 146 IS patients reported 77.8 and 94.2% sensitivity and specificity distinguishing the two stroke subtypes using a cut-off value of 0.03 g/l within 6 h of diagnosis (43).

Similarly, Katsanos et al. reported that in samples from patients presenting within 6 h from symptoms onset, significantly raised median plasma GFAP concentrations detected in ICH vs. IS, stroke mimetics, and controls. A cut-off of 0.43 ng/mL provided the best threshold for differentiation between ICH and AIS (sensitivity of 91% and specificity of 97%). They also described that the best timing of sampling to allow optimal differential diagnostic between IS and ICH was in the second hour from symptom onset (44).

A meta-analysis including nearly 1,300 patients confirmed the potential of measuring GFAP in the blood in the early phase of stroke (samples drawn <3 h from symptoms onset), to discriminate IS, ICH and mimics. Interestingly, there was no significant difference in diagnosis accuracy when patients were classified in three subgroups according to time of sampling (0–60, 60–120, 120–180 min after stroke onset) (82).

More recently, in a smaller meta-analysis including 340 patients (236 acute IS and 104 ICH) from four studies, Cabezas et al. confirmed that standardized levels of GFAP blood levels were significantly elevated in ICH compared with IS. Here again, the analysis showed no correlation of GFAP concentration with time of sampling (83).

Controversially, it was previously reported that when measured early (<1 h after stroke onset), serum GFAP did not distinguish ICH and IS (80).

Nevertheless, GFAP measurement is not part of routine clinical practice. The results described above need to be replicated by different groups in larger studies with standardization of detection methodologies and diagnostic cut-offs. This is especially important with respect to the differentiation of stroke mimics as GFAP has also been found in the serum from high-grade glioma patient (84) and is considered as a potential biomarker for diagnosis of traumatic brain injuries (85) which are both potential stroke mimics. Ideally GFAP sensitivity and specificity to distinguish hemorrhagic from ischemic stroke would need to be consistent across time, especially in the earliest time point from symptoms onset when the distinction is most important prior to initiation of thrombolytic therapy. If this is not the case, it will constitute a major barrier for therapeutic triage, especially in a prehospital setting, where timing of stroke evolution may still be uncertain. Refinement of GFAP measurement technologies and application strategies will be required.

### NSE

Serum concentrations of NSE (neuron-specific enolase) have been reported to be as significantly raised in stroke patients compared to controls and to correlate with infarct size and stroke symptom severity (75, 86–88). Serum NSE levels assessed prospectively within 4.5 h of IS symptom onset in rt-PA threated patients (*n* = 67) correlates with NIHSS at 24 h (R = 0.342), and lower serum NSE levels and NIHSS scores were detected in patients with favorable neurological outcomes after 90 days (45).

Overall, NSE has a similar discriminatory profile to S100B (high specificity and low sensitivity) (71–73, 88). This may in part be due variable kinetics of release, sometimes peaking 24 h after stroke (89, 90). Interestingly, Kim et al. showed that IS patients identified as having a second peak of serum NSE (20% of the studied population) were more at risk of developing hemorrhagic transformation (OR = 6.8) (90). Therefore, while NSE is not currently recommended for the diagnosis of acute stroke it may still have clinical potential.

### MMP-9

Expectations have been high for MMP-9 (matrix metalloproteinase-9) as a stroke diagnostic biomarker because of its role in response to brain injury *via* its involvement in extracellular matrix degradation. MMP-9 concentrations measured acutely have been linked increased to infarct size, worse neurological outcome, and complications of hemorrhagic transformation (46, 91–96). Serum MMP-9 concentrations ≥140 ng/ml were shown to predict hemorrhagic transformation in rt-PA treated ischemic stroke patients (sensitivity 92%, specificity 74%) (46). Six other studies confirmed the correlation between MMP-9 concentration and increased bleeding risk after rt-PA (96). Similarly, Barr et al. identified an association between elevated serum concentrations of MMP-9 and blood brain barrier disruption which is a key feature of hemorrhagic transformation (97). However, the rise of MMP-9 is not specific to ischemic stroke, moreover its concentration is reported to peak at 24 h post stroke (96), too late for making decisions about thrombolysis, and standardization of MMP-9 measurements and experimental replication are still required.

### NMDA-R

Autoantibodies to the glutamate NMDA-R (N-methyl-D aspartate receptors; NR2A/NR2B subunits) associated with neurotoxicity are elevated after stroke and distinguish IS patients (*n* = 31) from controls 3 h after symptoms onset with 97% sensitivity and 98% specificity (47). In a different cohort, plasma levels of NMDA-R NR2A were also shown to be elevated in ischemic strokes when there was no difference observed in patients with cerebral hemorrhage in comparison to controls (98). Criticism for the potential use of NMDA-R antibodies for the diagnosis of ischemic stroke were raised as NMDA-R antibodies have also been detected in patients with prior stroke hypertension, atherosclerosis, epilepsy, systemic lupus erythematous, and encephalitis (47, 98–100). Nevertheless, more recently, NR2 peptide (a product of degradation of NMDA-R) in blood has been reported to distinguish IS from stroke mimetics, patients with vascular risk factors and controls with 92 and 96% sensitivity and specificity, respectively (48). On the negative side, NR2 levels might not be increased in lacunar and small cortical strokes (48). Interestingly, detection of NMDA-R NR2A antibodies and NR2 concentrations might have a temporal profile after ischemic stroke (with a peak after 12 h) (48, 98) that might contribute to pinpointing a patients stage of stroke evolution but these results need to be validated in specifically designed studies.

### Apo-lipoproteins

Some members of the apo-lipoprotein family have also been tested as potential biomarkers for IS diagnosis. Apo C-I and Apo C-III concentrations were found to be increased in IS compared to ICH within 6 h of symptom onset and both were reported to have the potential to discriminate IS from ICH. For Apo C-III this was achieved with 94 and 87% sensitivity and specificity, respectively (49). A panel of nine apo-lipoproteins was tested as a tool to distinguish IS and ICH patients within the first week after symptom onset using a mass spectrometry assay. Apo C-I and Apo C-III reported to provide the best classification power as individual markers but combining Apo C-III and Apo A-I provided the best discrimination overall (AUC = 0.92) (101). Unfortunately, these results were not confirmed by Walsh and colleagues who looked at a broader panel which included paraoxonase-1, MMP- 3 and 9 and Apo A-I, C-I, and C-III for their ability to distinguish between IS, ICH patients and controls on blood samples obtained within 12 h of symptom onset. In this cohort, the levels of Apo A-I, Apo C-I, and paraoxonase-1, were shown to be lower in IS than in ICH patients with the other candidates having no discriminatory value (102). It is intriguing to speculate that this stark difference might be accounted for by a temporal component to the expression profile that might itself be useful.

Others have taken a ratio-metric approach to the use of apo-lipoprotein family members as stroke biomarkers. As et al. reported that Apo B concentrations and the Apo B/Apo A-I ratio were significantly elevated while levels of Apo A-I was significantly decreased in IS patients compared to controls. All three-potential biomarker tests were reported to have a high specificity and sensitivity to discriminate stroke patients (between 86 and 98%) (50). The Apo B/Apo A-I ratio has also been associated with early neurological deterioration in large artery atherosclerotic stroke, this was not found for other stroke subtypes (103).

### Others

Other less well-studied candidates may also have merit. For example, Allard et al. described first in 2005 the potential of PARK7 and NDKA (nucleoside diphosphate kinase A) as biomarkers for stroke diagnosis as their plasma concentrations increased early after symptom onset (29, 104). However, their specificity and sensitivity as markers were dependent on the diagnostic cut-off values used (104) and the results still need to be replicated. Tulantched et al. later specified that PARK7 seemed to have a better prognostic value than NDKA, both in sensitivity and specificity. Once more, collection time in this prospective study was late after stroke onset with a median of 17 h (51).

More recently, a prospective study found that glycogen phosphorylase isoenzyme BB measurements were able to discriminate between 172 IS and 133 controls with 93% sensitivity and specificity (cut-off of 7.0 ng/mL and sample drawn within 12 h of onset) (52). Nevertheless, glycogen phosphorylase which metabolizes glycogen to provide glucose-1-phosphate to restore energy stores has also been identified as a potential marker of ischemic myocardial injury (105, 106).

A meta-analysis interrogating over 130 biomarkers published by Hasan et al. in 2012, concluded that C-reactive protein (CRP), P-selectin and homocysteine were the only three biomarkers able to significantly differentiate ischemic stroke from healthy patients (28). Nevertheless, once more, these three molecules have a low specificity for ischemic stroke and therefore preclude their diagnostic use in acute stroke situations.

A systematic review performed by Misra et al. identified 10 single biomarkers and seven biomarker panels with a potential for differentiating IS and ICH. Once more, GFAP appeared to perform well, either as a single marker or in association either with the Activated Protein C- Protein C Inhibitor Complex (APC-PCI) or with the Retinol Binding Protein 4 (RBP4). Nevertheless, because the time of sampling was outside of the time window for practical acute stroke intervention (31), their clinical utility is still unclear. In another systematic review, Monbailliu and colleagues identified a different pairing of diagnostic biomarkers for consideration. BNP and S100 were the only two blood-based proteins biomarkers in their study that could differentiate IS from ICH, stroke mimetics and healthy control subjects (32).

The most recent meta-analysis published in 2020 analyzed 25 biomarkers across 40 studies and over 5,000 IS, 750 ICH, 550 mimics, and 1,770 healthy controls on samples collected within 24 h of symptoms onset. BNP, MMP-9, D-Dimer were identified to significantly differentiate the different patient groups while GFAP was successful to differentiate IS from ICH within 6 h. S100B, caspase-3 and NSE only distinguished IS from stroke mimics. Nevertheless, the authors highlighted that 67% of the studies included had only moderate study quality suggesting the need for further well-conducted studies (107).

While these markers all offer promise as diagnostic aids, until larger validation studies tease out the reproducibility of diagnosis, specificity in different patient groups and the role of sampling window in the value of the measurements, the current level of uncertainty does not recommend their immediate clinical use.

## Biomarkers of Disease Progression

In addition to the previously mentioned MMP-9, NSE, and S100B, other molecules are linked with increased bleeding risk after IS. Plasma levels of c-Fn (cellular-fibronectin), which reflect vascular damage, have been associated with the development of hemorrhagic transformation following t-PA use (108). When evaluated in a second cohort of 27 subjects, serum c-Fn ≥3.6 μg/ml identified hemorrhagic transformation with a sensitivity of 100% but specificity of 60% (46). Combining c-Fn with MMP-9 allowed detection of hemorrhagic transformation with 92% sensitivity, 87% specificity, and a positive predictive value of 41% (46). Reduced levels of PAI-1 (plasminogen activator inhibitor) and higher levels of TAFI (thrombin-activated fibrinolysis inhibitor), two endogenous fibrinolysis inhibitors, have been associated with symptomatic intracranial hemorrhage after thrombolysis therapy. When combined, PAI-1 level <21.4 ng/ml and a TAFI level >180% predicted symptomatic intracranial hemorrhage after rt-PA (sensitivity and specificity of 75 and 97.6%, respectively) (54).

Several biomarkers have been associated with early neurological deterioration (END). This has been defined as neurological worsening between 48 and 72 h after admission and occurs in one third ischemic stroke patients (109). Cytotoxic mechanisms mediated by glutamate, nitric oxide, and cytokines and endothelial-leukocyte adhesion molecules have been proposed as mediators of progression of tissue damage (110).

High plasma glutamate concentrations have been correlated with neurological worsening and infarct growth at 72 h after stroke onset (55, 110). Plasma glutamate concentrations of >200 μmol/l on admission have a positive predictive value for neurological deterioration at 48 h after lacunar infarction of 67% (55). Plasma GABA levels <240 nmol/l on admission also had a positive predictive value for neurological deterioration at 48 h after lacunar infarction of 84% (55). Higher levels of inflammatory markers such as ferritin, IL-6 (interleukine-6), TNF-α (tumor necrosis factor-α) and ICAM-1 (intercellular adhesion molecule-1) were also shown to be associated with early neurological worsening (110–112).

Space-occupying brain oedema (also called malignant oedema), an early life-threatening problem in patients with large hemispheric stroke, has been shown to be predicted by an elevated plasma S100B level (>0.35 g/l) with a 75% sensitivity and a 80% specificity at 12 h after stroke and even more at 24 h (94 and 83% sensitivity and specificity, respectively) (39). c-Fn and MMP-9 concentrations have also been found to be significantly higher in patients with malignant MCA (m-MCA) infarction than in controls. c-Fn concentrations of >16.6 μg/ml provided a 90% sensitivity and 100% specificity with 89 and 100% negative and positive predictive values, respectively, for prediction of m-MCA infarction (53).

While more work is needed to determine precisely when in a patient's clinical course these measurements first provide valuable information about that individual's likely outcome, their generally high sensitivity and specificity suggest they will find clinical utility.

## Biomarkers Panels

To better account for the molecular complexity of the ischemic cascade and increase the sensitivity and specificity of biomarkers as diagnosis tools, many researchers have also investigated biomarker panels, evaluating multiple molecules simultaneously instead of looking for a single biomarker. In a systematic review, Whiteley et al. identified seven panels of biomarkers tested as ischemic stroke diagnostic tools. The main criticisms were that the multimarker panel studies did not provide regression equations for stroke prediction and that a variety of cut-off values were used for the same biomarker. Moreover, the sample collection time points usually occurred outside the window where treatment was possible (29).

Reynolds et al. assessed plasma from 223 stroke patients (including IS, ICH and subarachnoid hemorrhage) and 214 healthy controls for more than 50 serum biomarkers using ELISAs (enzyme-linked immunosorbent assay). The combination of S100B, B-type neurotrophic growth factor (BNGF), von Willebrand factor (vWF), MMP-9, and monocyte chemotactic protein-1 (MCP-1) provided diagnosis of stroke within 12 h after symptom onset with a 91% sensitivity and a 97% specificity (56). A related panel of S100B, MMP-9, vWF, and vascular cell adhesion molecule (VCAM) studied by the same group of researchers in 65 suspected ischemic stroke patients and 157 controls within 24 h of symptoms provided a sensitivity and specificity of 90% (57).

In 130 patients with acute focal neurologic deficits admitted within 6 h of onset of symptoms, a panel including D-dimer, CRP, B-type natriuretic protein (BNP), MMP-9, and S100B was predictive of ischemic stroke with sensitivity and specificity of 81 and 70%, respectively (58). While less specific and sensitive than the preceding panels, its time window of application is more appropriate to the acute stroke setting (6 vs. 24 h). However, when the same panel of markers, excluding CRP, was tested in a prospective multicenter trial of more than 1,100 patients who presented with symptoms suggestive of stroke, a 86% sensitivity and 37% specificity were achieved for distinguishing stroke from mimics in the first 24 h after symptom onset (59).

In a study published in 2011, Montaner et al. tested, in an ED setting, a panel of blood biomarkers including CRP, S100B, MMP-9, a soluble receptor for advanced glycation end products (sRAGE), D-Dimer, brain natriuretic peptide (BNP), caspase-3, neurotrophin-3, chimerin, and secretagogin. They identified levels of caspase-3, D-dimer, sRAGE, chimerin, secretagogin, and MMP-9 as independent predictors of stroke vs. mimics. Moreover, they reported a predictive probability for identifying stroke of 99.01% by combining set cut-off values of these six biomarkers (60). The same team have also demonstrated, in a cohort of 915 stroke patients, that just S100B and sRAGE, could distinguish between IS and ICH with an AUC of 0.76 for blood samples obtained within 3 h after symptom onset. This was confirmed in blood samples obtained within 6 h of symptom onset (61).

More recently, measurement of retinol binding protein 4 (RBP4) (with a cut off value >61 g/ml) and GFAP (with a cut off value of <0.07 ng/ml) was shown to distinguish IS from ICH with a specificity of 100% in a cohort of 38 IS and 28 ICH samples (62).

In the STROKE-CHIP study, a prospective multicenter study of over 1,300 patients, published in 2017, Bustamante et al. studied a panel of 21 biomarkers selected from prior studies and published literature (including S100B, cFn, NSE, MMP-9 e.g.) on blood samples collected immediately upon arrival of patients presenting within 6 h after symptom onset. None of these biomarkers were able to provide an accurate hyperacute differential diagnosis of stroke (113).

While adding complexity to the laboratory work required, panels of markers appear to have the potential to offer significant improvements in specificity and sensitivity. However, further validation is still clearly required.

## The mRNA Revolution

The development of oligonucleotide microarray techniques, and more recently RNAseq, opened new perspectives in the quest for discovery of specific acute stroke biomarkers. These techniques allow unbiased investigation of the entire transcriptome as RNA shed from damaged or communicating cells, or contained within the cells of the immune system, the body's own “first responders” to injury. In addition, changes in mRNA expression occur very quickly often before changes of protein expression can be detected (114). This suits perfectly the acute ischemic stroke setting where “time is brain.”

Tang and colleagues reported a blood genomic response specific to ischemic stroke on blood samples collected at 24 h from rats subject to MCAo, sham surgery, and naïve controls. Twenty five genes were shown to be significantly (more than 2-fold) over expressed in rat blood 24 h after induction of ischemia while 98 had decreased significantly in comparison to controls (115).

Using blood samples collected from 20 patients with ischemic stroke and in 20 controls and stratified for sampling time (<24 h *n* = 7, 24–48 h *n* = 10, and >48 h *n* = 3), Moore and colleagues found that, after correction for multiple comparisons, 190 genes were differentially expressed (comparing stroke and control). Moreover, a panel of 22 genes identified as coming from peripheral mononuclear cells differentiated ischemic stroke from controls with 78 and 80% sensitivity and specificity, respectively (63).

When bloods were sequentially collected within 3 h, at 5 h and at 24 h from eight controls and 15 ischemic stroke patients [initially enrolled in the Combination approach to Lysis utilizing Eptifibatide And Recombinant tissue-type plasminogen Activator (CLEAR) trial], 104 genes were identified to have a 1.5-fold change between ischemic stroke and controls at 3 h, 1,106 at 5 h and 906 at 24 h. An 18-gene panel distinguished between ischemic stroke and controls with accuracy in 75% of the cases or more at all 3 different time points (64). Genes included in this panel reflected the involvement of inflammation in the ischemic pathway but were different from those identified by Moore.

When samples from the CLEAR trial were used to explore RNA expression after different ischemic stroke etiologies, 77 genes showed at least a 1.5-fold change in expression between large vessel occlusion and cardioembolic strokes. Twenty three of these genes could distinguish these etiologies with >95% sensitivity and specificity (65).

However, when RNA isolated from the peripheral blood mononuclear cells of acute ischemic stroke patients, stroke survivors and patients with acute traumatic brain injury was analyzed (cohort *n* = 15–20, sampling time: 24–27 h after event onset), no significant differences in single genes expression were identified between these groups. Nevertheless, expression of PDE4D (phosphodiesterase 4 D), an enzyme metabolizing cyclic adenosine monophosphate in inflammatory cells, was significantly different between acute ischemic stroke patients and healthy controls with cardiovascular risk factors (116).

A retrospective case-control study of 39 ischemic stroke patients and 25 controls (sampling time 10 ± 6.5 h), identified nine genes whose expression was significantly different in stroke patients and involvement of toll-like receptor signaling in the ischemic cascade (117). Five of these nine genes; MMP9, ARG1, CA4, LY96, and S100A12, had previously been reported as specific for stroke (64).

In a larger cohort of 194 blood samples collected at 3, 5, and 24 h after stroke from 76 patients with acute IS, a 40-gene panel distinguished cardio-embolic from large vessel strokes with >95% sensitivity and specificity. In addition, a 37-gene panel was identified to be able to differentiate atrial fibrillation from non-atrial fibrillation causes of cardioembolic stroke with >90% sensitivity and specificity (66).

Zhan and colleagues took a different approach and compared TIA with ischemic stroke. In rats they showed that only brief focal ischemia was needed to induce the majority of changes caused by ischemic stroke (118). When the same group compared the blood expression profiles of TIA patients (*n* = 26) and control subjects with vascular risk factor but without symptomatic cardiovascular disease (*n* = 26), they identified 449 genes that distinguished between the two groups. Thirty-four genes separated TIAs from controls with 100% sensitivity and specificity. In addition, two different patterns of gene expression were identified by cluster analysis for the TIA patients suggesting a heterogeneous response to the event between patients and a possible relation with a higher risk of stroke (67).

These findings were soon tested in a bigger cohort by Jickling and colleagues. In 164 blood samples collected within 72 h of symptom onset from stroke, TIA and control patients, 145 genes were differentially expressed between TIA and controls and 413 genes were significantly different between IS and controls. More importantly, 74 of the 145 genes identified in the TIA group were also found in ischemic stroke patients. Twenty six of these 74 common genes were used as a panel to distinguish stroke and TIA from controls with 89% sensitivity and specificity. Pathways analysis revealed that the genes common to stroke and TIA were involved in innate and adaptive immune systems activation involving B-cells and granulocytes (68). Unfortunately, the authors did not reveal the composition of their 26-gene panel, so comparison with the 34-genes panel identified earlier by Zhan et al. is not possible.

Jickling et al. also evaluated the gene expression profile of lacunar strokes. In a cohort of 30 lacunar and 86 non-lacunar strokes (with blood sampling within 72 h of stroke onset), they identified a 41 genes discriminating lacunar and non-lacunar stroke with >90% sensitivity and specificity (69).

In 2012, Oh et al. performed microarray analysis on blood samples collected from 12 ischemic stroke patients and 12 controls (sampling time 12.7 ± 5.3 h after stroke onset). They identified 88 transcripts with a 1.5-fold change in ischemic stroke compared to controls and 11 transcripts with 2-fold difference (including MMP9, Il1R2). Then, they validated the expression of the three most differently expressed genes (MMP9, Il18RAP, and GNLY) by quantitative polymerase chain reaction (qPCR). In another cohort of 120 ischemic stroke patients and 82 controls (sampling time 10.4 ± 9.7 h). MMP9 concentrations measured using ELISA were significantly greater in IS compared to controls but did not to correlate with infarct volume (119).

When quantitative PCR was used validate the expression profiles of 40 candidate biomarkers identified in previous studies (63, 64, 117) in 18 ischemic stroke patients and 15 controls (median time of blood sampling 36 h), 16 genes were significantly upregulated in ischemic stroke patients in comparison to controls. Six gene clusters were reported to discriminate between stroke and controls and one of them, containing seven transcripts, was reported to show high accuracy for stroke classification (120).

In common with the candidate protein biomarker studies described earlier, the investigators for the transcriptome studies summarized above have tended (samples from the CLEAR trial are an obvious exception) to perform analyses relatively late in stroke evolution, when diagnosis is generally already certain and decisions about therapy already made. Moreover, there has been little emphasis on distinguishing ischemic and hemorrhagic stroke, or identifying genes that might identify a heightened risk of bleeding. These gaps in the analysis are surprising. Array technologies also lend themselves to collaborative re-analysis, indeed many publishers stipulate that array data be made freely available. It is therefore also surprising that pooled analysis of the available data has not yet been performed.

## Biomarkers of a Stroke Clock

As mentioned above, stroke biomarker discovery has rarely focused on early temporal change, despite the dynamic characteristics of stroke. The possibility that changes in expression of candidate biomarkers with time might help predict stroke evolution and act as a biological stroke clock which could allow more patients to be recruited to thrombolysis is largely unstudied.

In serial blood samples collected at 3, 6, 12, 18, 24, 48, 72, 96, and 120 h after onset of stroke symptoms, NSE concentration, measured by immune-assay, rose in the first 2–3 h, then fell until 12 h before a second elevation that was maintained until measurement ended on day 5. Tau concentration showed a continuous increase from admission onward (87).

During a study evaluating the diagnostic performance of 29 pre-selected molecules within the therapeutic window for thrombolysis in 103 stroke and 132 control patients, glutathione S-transferase-π (GST-π), an enzyme providing protection against oxidative stress, was the most significantly elevated molecule in stroke patient blood. Importantly, GST-π measurement allowed the discrimination of early (<3 h) and late (>3 h) presentations of stroke in 90% of the cases with a cut-off value of 17.7 μg/l. Indeed, GST-π concentration was almost immediately after stroke with increases detected within 3 h after symptom onset and within 1 h in some. Importantly, GST-π concentration decreased rapidly after 3 h reaching a concentration close to normal levels by 6 h after stroke symptoms onset. When GST-π was measured in a cohort of thrombolysed stroke patients (blood collected within 3 h after stroke onset, *n* = 100), its concentration was elevated above the threshold of 17.7 μg/l in 98% of the cases. A similar but less striking pattern was observed for PARK7and NDKA (70).

Conversely, in plasma samples collected at 12, 24, and 48 h after symptoms onset in 39 patients with ischemic stroke, while MMP-9 concentrations were greater in stroke patients than the reference interval for healthy controls, no significant changes were reported over time (95).

Others have collected human blood samples sequentially in the same patient early after stroke, but the analysis focused on creation of a diagnostic tool able to differentiate IS patients from controls and blood samples were not collected within 3 h after the ischemic event (results presented previously) (64).

To date, these are the only investigations identifying blood born biomarkers with a potential to contribute to development of a stroke clock and a potential ability to discriminate eligible vs. ineligible patients for reperfusion therapy.

Nevertheless, clinical trials for the discovery of diagnostic stroke biomarkers suitable for use in the hyperacute phase of the disease are underway. Some of these trials hope to identify biomarkers that will aid stroke diagnosis on admission to the clinic.

The multicenter, observational Biomarkers of Acute Stroke Etiology (BASE) study aims to identify biomarkers defining acute IS etiology and is recruited patients presenting within 24 h of symptom onset. Blood samples are being obtained on arrival and 24, and 48 h later, and gene expression profiling is being used to identify biomarker candidates of stroke (121).

Results of the innovative Blood And Clot Thrombectomy Registry And Collaboration (BACTRAC) trial could also lead to new findings in the stroke biomarker field. Fraser et al. aim to collect intracranial thrombus material and arterial blood collected before, after and during mechanical thrombectomy to allow gene expression and proteomic analysis of the early human molecular response to ischemic stroke (122).

The Helsinki Ultra-acute Stroke Biomarker Study even sampled in a pre-hospital setting *via* blood samples taken by emergency medical service clinicians during transit to analyze GFAP and NR2 peptide levels explore novel markers. The recruitment phase is over but the study has yet to report on the primary outcomes (123).

## Conclusions

Improving in patient outcomes in acute stroke requires a rapid and accurate diagnosis of stroke and its subtypes. A biomarker that could differentiate between hemorrhagic and ischemic stroke or risk of subsequent bleeding would, in theory, permit widespread initiation of thrombolysis in the ambulance and save valuable time and brain tissue.

Markers of brain tissue damage, particularly the highly abundant glial structural proteins like GFAP and S100β and the matrix protein MMP-9 offer this promise but have not yet been systematically evaluated at the earliest time points which matter most. To date, other highly abundant structural proteins such as those characteristic of axons, dendrites, and synapses or oligodendrocyte processes have rarely been considered for this role with the exception of the NR2 degradation product of the NMDA receptor and PARK7 which has a specific anti-oxidant role.

Whether such molecules will be able to rule out stroke mimics which also damage the structure of the brain remains to be determined. In this regard, the circulating apolipoproteins (Apo A1, Apo C1, and Apo C111) and c-FN, PAI-1, and TAFI which may specifically react to the hematological changes of a hemorrhagic stroke or hemorrhagic transformation, respectively, require further study. Accurate prediction of poor outcome after stroke would help patients, their families and clinicians to make early and informed decision about choices between rehabilitation and palliative care.

The suggestion that autoantibodies to NMDA receptors might help in this task raises the question of whether their presence in ischemic stroke signifies previous undetected ischemic events and thus heightened stroke risk. Patients with acute minor IS or TIA are at risk of further occlusive vascular events, particularly recurrence of stroke (124, 125). Prognostic scores based on clinical characteristics observed when first assessed, such as the ABCD^2^ score (126), tend to predict early stroke recurrence risk but they do not discriminate perfectly between those individuals who will have a recurrent stroke and those who will not (127). Specific biomarkers which helped stratify this risk would be of considerable value but might be of little use in diagnosis of first ever stroke.

If selecting candidate biomarkers based on prior knowledge of involvement in stroke pathophysiology has yet to prove successful, the high costs of “omic” discovery strategies has limited the scope of their use and is still in its infancy. Developing panels of markers from either source and developing ratiometric approaches to analysis seem to offer the hope of significantly better specificity and sensitivity.

For both strategies, most measurements made to date have been performed later than the clinically critical thrombolysis and thrombectomy time window. Timing of biomarker measurement, particularly early when decision making is most important, requires urgent and systematic study. The kinetics of change may be revealing in their own right and, if a biomarker stroke clock can be constructed, might dramatically broaden the utility of thrombolysis and thrombectomy.

The recent discoveries in advanced cerebral imaging and the subsequent extension of time window for both thrombolysis and thrombectomy highlight that specific biomarkers of penumbra would be even more crucial than biomarkers of time for therapeutic decision making in the acute setting. Research combining imaging and biological biomarkers is needed.

The ultimate aim of the stroke biomarker research is the development of a point of care device. A quick and reliable bedside biomarker assessment would revolutionize the acute stroke management. It could potentially expedite the diagnosis of ischemic stroke by making the imaging step redundant and aid the clinical decision-making (even in a prehospital setting). It will reduce time from symptoms to initiation of reperfusion therapies. Specific biomarkers could also be used for pre-hospital stratification of important subgroups. Indeed, they might help identify patients with large vessel occlusion and facilitate direct access to comprehensive stroke centers and timely thrombectomy. Stroke biomarkers could help to resolve the mothership vs. drip and ship dilemma.

Most of the candidate biomarkers described in this review have been detected by what are best described as research tools (e.g., ELISA, Western Blotting, Mass Spectrometry, Gene array, RNASeq) which have inherently long lead times before a result might be available for a clinician to use. However, a range of assay systems are capable of providing results within minutes, both in a laboratory and point of care setting.

Good examples of rapid assays that could be adapted for stroke biomarker detection include a range of widely used clinical tests based on the principles of sandwich ELISA, in which a target protein is first captured to the surface of the assay device and then detected by a second antibody bearing an easily detected label (128). Perhaps the best known of these are pregnancy tests that detect human chorionic gonadotrophin within a few minutes of sample application (129). Numerous point of care immune assays for biomarker detection are currently under evaluation (130).

Other assay methodologies also have potential for rapid detection of stroke biomarkers. For example, blood glucose can be detected even more rapidly (5 s) by using electrochemical detection of the reaction products of glucose oxidase activity (131). Miniaturization now also makes highly sensitive and selective and rapid analyte detection by a range of mass spectrometry protocols possible, even at the bedside (132). Even nucleic acid biomarkers can now be detected within minutes, with recent publications reporting completion of 30 qPCR cycles within 54 s (133), certainly fast enough for stroke diagnostics if the promise of portable devices that might be used at the bedside (134) are realized. Moreover, nanotechnology offers the promise of highly multiplexed biosensors capable of rapid simultaneous analysis of large panels of biomarkers (135), an important consideration if multiple analytes must be assessed to provide stroke diagnosis and prognosis.

However, it has to be concluded that none of the candidate markers described in this review have entered routine clinical use despite their obvious promise. More work is required before lives can depend on such measurements.

## Author Contributions

DH conceived the manuscript. MD wrote the original draft. MD, GD, SD, HD, and DH edited the document. All authors contributed to the article and approved the submitted version.

## Conflict of Interest

The authors declare that the research was conducted in the absence of any commercial or financial relationships that could be construed as a potential conflict of interest.
